# Anthropogenic Influences on Conservation Values of White Rhinoceros

**DOI:** 10.1371/journal.pone.0045989

**Published:** 2012-09-27

**Authors:** Sam M. Ferreira, Judith M. Botha, Megan C. Emmett

**Affiliations:** 1 Scientific Services, South African National Parks, Skukuza, South Africa; 2 Rhino Action Group Effort, Johannesburg, South Africa; Australian Wildlife Conservancy, Australia

## Abstract

White rhinoceros (rhinos) is a keystone conservation species and also provides revenue for protection agencies. Restoring or mimicking the outcomes of impeded ecological processes allows reconciliation of biodiversity and financial objectives. We evaluate the consequences of white rhino management removal, and in recent times, poaching, on population persistence, regional conservation outcomes and opportunities for revenue generation. In Kruger National Park, white rhinos increased from 1998 to 2008. Since then the population may vary non-directionally. In 2010, we estimated 10,621 (95% CI: 8,767–12,682) white rhinos using three different population estimation methods. The desired management effect of a varying population was detectable after 2008. Age and sex structures in sink areas (focal rhino capture areas) were different from elsewhere. This comes from relatively more sub-adults being removed by managers than what the standing age distribution defined. Poachers in turn focused on more adults in 2011. Although the effect of poaching was not detectable at the population level given the confidence intervals of estimates, managers accommodated expected poaching annually and adapted management removals. The present poaching trend predicts that 432 white rhinos may be poached in Kruger during 2012. The white rhino management model mimicking outcomes of impeded ecological processes predicts 397 rhino management removals are required. At present poachers may be doing “management removals,” but conservationists have no opportunity left to contribute to regional rhino conservation strategies or generate revenue through white rhino sales. In addition, continued trends in poaching predict detectable white rhino declines in Kruger National Park by 2016. Our results suggest that conservationists need innovative approaches that reduce financial incentives to curb the threats that poaching poses to several conservation values of natural resources such as white rhinos.

## Introduction

Iconic species that have valuable assets, such as horns or pelts, suffer greatly from human persecution [1]. African mega-herbivores epitomize threats posed by such human persecution. The use of ivory, for instance, has for centuries influenced elephant *Loxodonta africana* abundance and behavior [2], while perceptions about medicinal properties of rhinoceros (rhino) horn [3] made several rhino species lucrative targets [4]. Modern commercialization as well as technological advances facilitated the exploitation of biological resources [5]. The end result is that, globally, biological exploitation is a key driver of declines in a range of taxa [6]. Most notably are Africa’s large mammals, even in protected areas [7].

Species with specific features are likely to be most at risk when biological exploitation is commercial. These include non-renewable assets (*e.g.* elephants with ivory), small populations (*e.g.* tigers *Panthera tigris*), and slow life-histories (*e.g.* rhinos). Potential threats to the persistence of white rhinos (*Ceratortherium simum*) in the Kruger National Park, a population stronghold for this species [4], reflect some of these challenges. Poaching of white rhinos has increased dramatically since 2006 [8] most likely fueled by the recent increase in the value of rhino horn [9]. International trade in rhino horn remains prohibited, while trade within most countries is also illegal [10]. Even so, the value of rhino horn is likely to provide complex incentives to criminal elements [11].

In addition, conservation agencies seek to restore degraded ecological processes. If this is not possible, they seek to mimic outcomes of impeded ecological processes [12] such as the influence that resource distribution has on the spatial use, associated intensity of landscape use and cascading ecological effects of mega-herbivores [13]. Resource distribution and availability can be altered through fences and water provisioning [14] which may have detrimental effects on conservation objectives through impeded spatial and demographic responses of mega-herbivores. Conservationists remove excess individuals generated by impeded population responses and have an option of using these for the establishment of other populations or, alternatively where wildlife can be traded, making these available for sale. Reconciliation of apparently contrasting biodiversity and financial objectives in such a way provides revenue that plays a key role in sustaining conservation areas [15]. Managers of Kruger National Park, a protected area with numerous additional water points [16], use white rhinos within this framework to achieve biodiversity objectives and generate conservation revenue. Illegal removal of rhinos through poaching may also thus impede on other objectives that conservationists seek to achieve.

Although the driver of rhino poaching is primarily economic through the demand and supply ratio that determines the rhino horn market value and hence poaching incentive [11], the consequences are varied. In the first instance is the threat that rhino poaching poses to the persistence of rhinos. The second consequence is the threat to other potential values such as the value of rhinos as a live commodity, the trade of which is legal [10]. Finally, society at large may experience variable consequences. In some instances societal degradation may result through associated organized crime [8], but for the end-user of horn [3], quality of living may increase through the placebo effect.

Within southern Africa, white rhinos are iconic and carry primarily two values – a purist conservation value, and a legal financial value (live rhino trade as well as rhino hunting). Here we evaluate threats posed by the present trends in poaching to the persistence of white rhinos in Kruger National Park, the largest population in the world. We also evaluate the potential consequences on contributions to populations elsewhere as well as traditional revenue generation through game sales. We then make suggestions on addressing these challenges in the short, medium and long terms.

## Methods

### Ethics Statement

The study made used of standard approved techniques to survey large mammals and did not require ethical approval since no animal was handled in the research.

### Study Area

**Figure 1:**
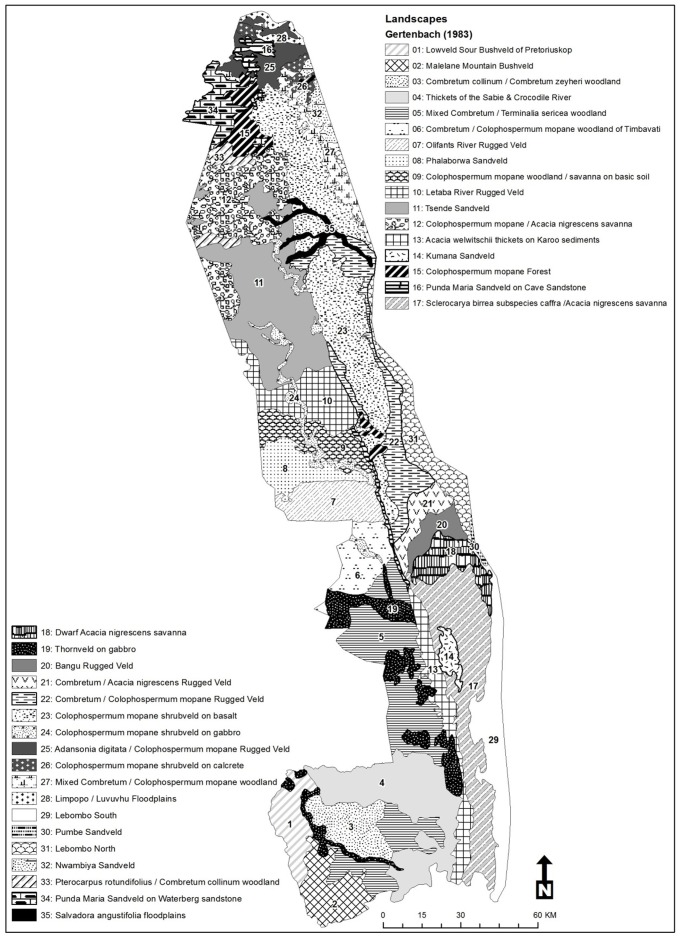
Kruger National Park and surrounds indicating landscapes as well as regions used to define population growth rates at different scales.

Kruger National Park is situated in the low-lying savannas of the eastern parts of the Limpopo and Mpumalanga provinces of South Africa (Fig.1). The Park covers an area of 19 485 km^2^, has mean annual rainfall that varies from 750 mm in the south to 450 mm in the north falling mostly during October to March [17]. Soils are derived from granite and gneiss deposits in the west and nutrient-rich basalts in the east. Karoo sediment is present where granite and basalt soils join [18].

Wooded savanna, with *Sclerocarya caffra* and *Acacia nigrescens* dominating the tree canopy comprises the bulk of the southern basalts, while mixed *Combretum spp.* and *Acacia spp.* dominate the southern granites. In the north *Colophospermum mopane* dominates all substrates. [19]. The underlying geology and vegetation defines 35 landscape types (Fig. 1, [20]).

### Conceptual White Rhino Management Model

Kruger National Park has an extensive ecological management history [21], most of which influenced resource distribution or access to resources. For instance, traditional landscape interventions interfere with vital rates of populations and fall into three categories: 1) those that affect dispersal such as fences and water provision [16], [22]; 2) those that affect survival such as culling, removals and water provision [23]; and 3) those that affect fecundity such as contraception [24] and culling through reduced density-depend effects on birth rates [25]. Conservationists can address such effects of historical legacies by restoring spatial and temporal limitations and/or mimicking the effects of spatial and temporal limitations when restoration is constrained for several reasons [12]. This reflects a paradigm of the flux of nature which upholds that heterogeneity enhances diversity which enhances resilience [26].

**Figure 2:**
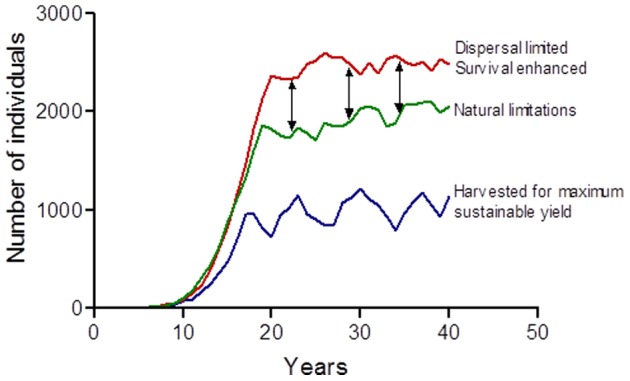
Conceptual population sizes across time. Note that a herbivore may stabilize at different population sizes depending on conditions imposed *e.g.* naturally limited (green line), constrained (red line) and harvested for maximum yield (blue line). The arrows indicate the likely available numbers for economic gain that will also enhance biodiversity objectives which focus on mimicking processes that has been impaired by landscape constraints. Most important is the temporal variability after stabilization that is wide and non-directional.

Although conservationists in Kruger are attempting to restore the variability in resource availability (*e.g.* closure of waterholes), remaining constraints as well as population lag effects continue to generate adverse population responses particularly of mega-herbivores [27]. Mimicking the outcomes that result if these impeded factors were not present is what white rhino management in Kruger seeks to do. The mimicking effect could generate sources of rhinos for establishing populations elsewhere as well as provide opportunities to sell rhinos for financial gains. Herbivore populations may stabilize at different sizes depending on conditions imposed *e.g.* naturally limited, human altered through, for instance, landscape interventions, and harvested for maximum yield (Fig. 2). When landscape interventions have removed population limiting and regulating mechanisms, abundances may increase. Responding to the excess created by impeded ecological limiting and regulatory factors provides for gains that will also enhance biodiversity conservation objectives. Most important is the temporal variability in this scenario that is relatively large and non-directional.

Maximum sustainable yield models define *r_max_* of a population [28] and define the instantaneous harvesting rate (*H_MSY_*) at anytime during the year as 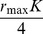 where *K* is an estimated equilibrium size around which a population may fluctuate. Harvesting a population at rate *H* is likely to result in population sizes lower than what natural limitations will result in. Mimicking process outcomes requires SANParks to remove fewer animals. For ecological management purposes SANParks thus remove rhinos at an instantaneous rate of 
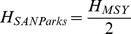
. Because estimates of *r_max_* have a statistical distribution, *H_SANParks_* will also have one. By drawing randomly out of the distribution and removing rhinos at that rate, SANParks induces variation.

Inducing spatial and temporal variation through managing numbers of a species may be enhanced through inducing source-sink dynamics [29]. Source-sink dynamics may lead to local instability, but regional stability [29], a feature desirable if conservationists wish to maintain persistent biodiversity. However, such strategies may lead to drifts in age structures that may carry long term consequences for the population specifically if removal of excess is selective [30]. These concerns are pertinent for the white rhino management model that Kruger managers adhere too. In addition, poaching can significantly impede the implementation of this model.

### Data Collection

We collated white rhino survey data for Kruger National Park from electronic databases (SANParks), unpublished reports and peer-reviewed publications. Fixed-wing based surveys covering 100% of Kruger National Park took place during 1960–1961, 1964, 1969–1993 as well as 1997. These total counts involved systematic low-level flying (≈300 feet above ground) with a light fixed-wing aircraft searching 64 blocks intensely and recording all rhinos encountered. During 1994 to 1996 similar approaches were used, but not all survey blocks were completed (SANParks, unpublished data).

From 1998 to 2010 counters used sample-based approaches [31]. With the exception of 2009, rhinos were recorded as part of the fixed-wing based annual herbivore survey of Kruger National Park each year. Sampling was based on flight paths with distance sampling estimating approaches [32] covering 15–22% of the Park. Stratified Jolly-Seber fixed-width estimating approaches using the same data resulted in 7.5–23.9% coverage of the Park. Note that transects had an east-west orientation and were spaced evenly across Kruger in a north to south configuration. Larger spacing leads to lower coverage and *vice versa*. The outcome is that all landscapes within Kruger had equal survey intensity relative to the extent of coverage in the Park.

We collated our final white rhino survey data set for 2009 from a black rhino (*Diceros bicornis minor*) block-based survey south of the Olifants River with coverage of 21.5% [33]. Counters also noted white rhinos in this survey. The survey comprised 3kmx3km blocks intensely searched from a helicopter observation platform.

Note that since 1998 spatially explicit records of white rhino survey data were kept which allowed us to focus on data since then in an attempt to understand landscape differences in white rhino dynamics. For the period from 1998 to 2011, we also collated records of poaching incidences (SANParks database, Corporate Investigation Services) as well as spatially explicit management removal records (SANParks database, Veterinary Wildlife Services) that included sexes and ages. Prior to 1998, we collated the total number of white rhino introduced or removed for ecological management reasons.

For 2009–2011, we annually defined the standing age distribution for white rhino during February and November each year. The survey targeted nine specific areas in the southern parts of the Kruger National Park. These areas had different histories of rhino removals for management purposes. We defined sinks (areas where rhinos have consistently been removed) and sources (areas directly surrounding sink areas) and controls (areas where rhinos were never removed and which are far away from sinks). The survey made use of helicopter-based search flights at a height of 350 feet flying at 60 knots and aimed to assign age (following [34] adapted to the diagrams of [35]) and sex to at least 100 individuals in each area.

### Data Analyses

We focused our analyses on data collated since 1998 when white rhino survey information was spatially explicit and poaching and removal statistics were well kept. For white rhino surveys conducted using fixed-wing aerial approaches (1998–2008 and 2010) we estimated population sizes in three ways. First, we used distance sampling approaches [32] that corrects for detection probabilities declining the further a rhino was from the flight path. Second, we used the same observations, but restricted our data to within 200 m either side of the flight path and applied a Jolly-Seber strip transect analytical approach [36]. In the third instance we allowed the strip to be 400 m on either side of the flight path and applied the Jolly-Seber strip transect analytical approach again. For all three analytical approaches, we obtained annual park-wide population estimates and 95% confidence intervals.

No formal white rhino surveys were conducted during 2009. White rhinos noted during the black rhino survey south of the Olifants River [33] allowed us to apply a Jolly-Seber strip transect analytical approach [36] that provided us with estimates of the number of white rhinos south of the Olifants River. Observers noted 89.8% of white rhino observations during 2008 and 90.6% of observations during 2010 south of the Olifants River in the fixed-wing based aerial surveys. We used the average of these two proportions to estimate a park-wide 2009 population size for white rhinos from the block-based survey south of the Olifants River.

To define a generalized trend for white rhinos in Kruger National Park since 1998, we checked how the confidence intervals of population estimates derived from each method of estimating abundances overlapped. We considered estimates as outliers for estimators that gave non-overlapping confidence intervals with the other estimators in a particular year. These outliers were subsequently excluded when defining the generalized trend. For estimators retained, we extracted 10000 random values for each year from the statistical distribution defined by the mean estimate and 95% confidence intervals. We combined these random values for all estimators in a specific year and calculated the mean as an estimate of the likely population size in a particular year. We also extracted the 2.5% and 97.5% percentile as estimates of the upper and lower 95% confidence limits respectively.

Following the definition of year-specific population estimates for 1998 to 2010, we expected two kinds of potential population models that may describe a generalized trend – an exponential model and a model considering some form of density dependence. We thus used maximum likelihood methods [37] to derive parameters for the following two models:
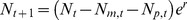
(1)

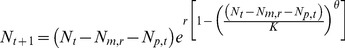
(2)where *N_t_* and *N_t+1_* are population size at time *t* and *t+1* respectively, *N_m,t_* and *N_p,t_* are the number of rhinos managers removed and poachers killed between time *t* and *t+1*, *r* is the exponential growth rate, *K* is the likely population size when the population will vary non-directionally and *^θ^* is a co-efficient that determines the shape of density dependence [38]. We used Akaike Information Criteria to evaluate which one of these two models is the most suitable [39].

To evaluate the historic anthropogenic effects we used the number of rhinos removed by conservation managers and poached between annual estimates to calculate what the likely population of white rhinos would have been at any time *t* during 1998 to 2010 if either or both of these anthropogenic removals were not present. For this purpose we used the instantaneous population growth (*r_t_*) at time *t* derived from 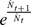
where  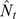 and 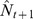 are the fitted population estimates from Equation 2, used the number removed and poached in the period from *t* to *t+1* and calculated how many extra rhinos would have been present at time *t+1*. These were added to 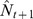 to provide estimates in the absence of management removals (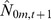), poaching (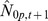) or both (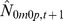). The process was repeated for each subsequent year from 1998 to 2010. We concluded that anthropogenic effects were significantly detectable when any of these predicted estimates were outside the confidence intervals of estimated population sizes from white rhino survey data.

To check effect of management removal and poaching on rhino population structure we pooled all data for sink, source and control areas noted during February each year (2009–2011), and estimated the sex-specific proportion of calves (0–4 years), sub-adults (5–6 years) and adults (7 years and older). We then calculated similar sex-specific proportions derived for that year from the data on rhinos removed for management purposes. We estimated the average sex-specific proportions for the standing age distribution of all three years in February and compared these to the average sex-specific age distribution of all three years’ removals. For the poaching effect, we only had data available for 2011 and we used a similar sex-specific comparison of poached age distributions with the standing age distribution of the population in 2011 only.

We anticipated that if management removals are key drivers of rhino population dynamics, sex and age structure should differ between sources and sinks. To check this we estimated sex-specific proportions of calves, sub-adults and adults like before for sinks and sources separately each year from 2009–2011. By comparing average sex-specific proportions calculated by combining all three years between sources and sinks we could evaluate this prediction.

Because different landscapes are likely to impose different resource limitations on white rhinos, conservation managers removed rhinos where logistically possible and poachers do not kill rhinos evenly across landscapes, we anticipated that these factors will vary across landscapes. Our observations were not numerous enough to extract landscape-specific estimates applying distance sampling analytical techniques [32]. Neither was poaching statistics spatially explicit prior to 2011. However, our analyses (see later) illustrated that poaching effects are not detectable at the population level. Management removal statistics, in contrast, were spatially explicit. Thus, for this part of our analyses we used only estimates derived from Jolly-Seber analytical techniques [36] applied to strip width data collated for 200m on either side of flight paths. This removed the detection effect that distance sampling identified to which the 400m strip width was vulnerable. We fitted exponential and equilibrium models as before for each landscape using maximum likelihood approaches [37] and used Aikaike Information Criteria [39] to choose which model fits the available data the best.

In addition, we calculated what the exponential population growth was in each landscape since 2006, the period when most aggressive removal of white rhinos by managers took place. We then asked how these growth rates associated with abundance, the ratio between abundance and predicted *K* for each landscape from the fitted equilibrium models, and the number of rhinos removed. We transformed the ratio (inverse) and number of rhinos (natural logarithm) to linearize the potential relationship with growth rate. We then used multiple linear regression analyses to evaluate several combinations of variables as potential explanations for variation in population growth rate between landscapes using model selection procedures as before [39]. Abundance serves as an index of potential statistical effects, specifically small populations, on population growth, while the ratio measure serves as an index of density-dependent population effects.

Finally, we evaluated the future potential consequences of sustained poaching trends for rhino population persistence as well as opportunities to contribute to other populations or generate revenue given the management model that SANParks adheres to. For this purpose we used *r_max_* and its confidence intervals derived for the best fit population model to define annual instantaneous removal rates *H_SANParks_* and its confidence intervals. At the same time, we fitted an exponential model to the proportion of rhinos poached annually since 1998. These two models predicted the number of rhinos required to be removed each year as well as the number of rhinos expected that would be poached respectively. We could then predict what the population would be if these events took place and from that prediction calculate the exponential growth rate at time *t+1* as 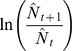. By simulating models 200 times using values drawn from the confidence intervals of parameters describing trends in poaching proportions and instantaneous management removal rates, we could estimate confidence intervals for the predicted number of rhinos removed, poached, as well as exponential growth rate at time *t+1*. We identified the year that poaching exceeds management removals (confidence intervals do not overlap) as the time when conservation value reflected as contributions to other populations of revenue gains through game sales, stops. We also identified the year when population growth at time *t+1* is significantly lower than zero (confidence interval is smaller than and excludes zero) as the time when rhino population persistence as a conservation value is at risk.

## Results

### Historic Trends

**Figure 3:**
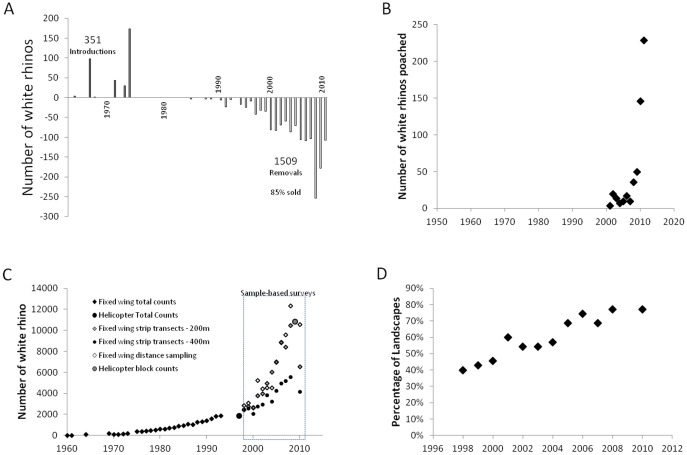
Historical trends in A) white rhino management, B) white rhino poaching, C) white rhino population surveys and estimates, and D) the presence of rhinos in different landscapes in Kruger National Park.

Park managers introduced 351 white rhinos between 1960 and 1972 and started to remove rhinos for donations to other conservation areas and zoological gardens during the mid 1980s (Fig. 3A). Since the late 1990s, a large fraction of the white rhinos removed was sold to generate conservation revenue. By the end of 2010, a total of 1402 white rhinos have been removed from the Park.

Even so, rhinos continue to colonize Kruger National Park with the percentages of landscapes on which counters noted rhinos continuing to increase from 1998 to 2010 (Fig. 3B). During this period counters were encountering rhinos in new landscapes at a rate of 1.19 (95% CI: 0.91–1.41) landscapes per annum. By 2010, 77.1% of the Park’s landscapes had white rhinos present.

Incidences of poaching were relatively low from the 1960s until a dramatic increase since 2006 (Fig. 3C). Well kept records since 1998 illustrate that poaching incidences increased exponentially per annum (y = 0.042e^0.616x^, *R^2^* = 0.89, *F*_1,12_ = 17.68, *p*<0.01). During 2011, 252 white rhinos were poached in the Park.

**Figure 4:**
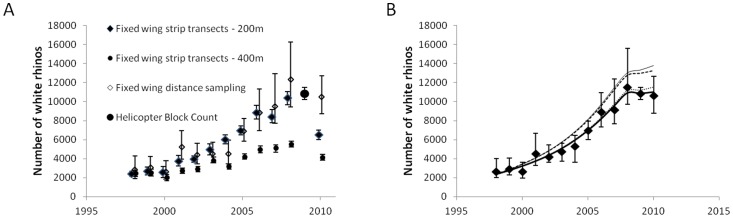
White rhino population estimates from 1998 to 2010 when sample-based estimates were used. We provide 95% confidence intervals (error bars) for different survey platforms and estimating techniques (A). Following estimator averaging, we present the generalized trend in white rhino population estimates (B). The solid thick line is the best fit model (see Table 1). We also present the predicted estimates if no removals or poaching took place (solid thin line), if no removals took place (thin broken line) and if no poaching took place (thin stippled line).

Since the 1960s the number of white rhinos in Kruger National Park has been increasing (Fig. 3D). This was also the case for different survey approaches and application of different estimators to survey data from 1998 onwards (Fig. 4A).

### Generalized Population Trend from 1998 to 2010

Jolly-Seber estimates derived from fixed-width strip surveys with strips 400 m wide on either side of the flight path were consistently lower than those derived from all other estimators (Fig. 4A). These were excluded and remaining estimator averaging suggested that the white rhino population in Kruger National Park increased from 1998 to 2008, but appears to fluctuate non-directionally since then (Fig. 4B) as the equilibrium model (Equation 2) was best suited to explain the available data (Table 1). During 2010 we estimated that 10621 (95% CI: 8767–12682) white rhinos lived in Kruger National Park.

**Table 1:** Population models for observed white rhino estimates in Kruger National Park.

	Exponential	Equilibrium
Model	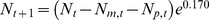	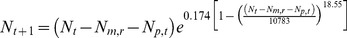
*RSS*	9754149	2818209
*R^2^*	0.92	0.97
*AICc*	181.07	172.73

We provide the residual sum of squares (*RSS*), coefficient of determination (*R^2^*) as well as Aikaike Information Criterium (*AICc*) as illustration of best model fit given the data.

### Anthropogenic Effects

If poaching and management removals did not take place, significantly more white rhinos would have lived in Kruger National Park (Fig. 4B). This effect, however, was only detectable during 2009 and 2010, when predicted estimates in the absence of anthropogenic factors were higher than the upper confidence limits of population estimates then. In the absence of both these factors, a mean estimate of 13794 rhinos (observed population size is 23.0% in reduction of potential population size) may have been noted during 2010.

Considering poaching and management removals separately resulted in only management removals having a detectable effect on white rhino population sizes during 2009 and 2010. The effect of poaching alone resulted in predicted estimates in the absence of poaching falling within the 95% confidence intervals of population estimates derived from white rhino survey data. In the absence of management removals, but with poaching present, a mean estimate of 13289 (20.1% reduction) may have been noted for 2010, while no poaching, but with management removals, would have resulted in a mean estimate of 11525 (7.8% reduction) white rhinos during 2010.

**Figure 5:**
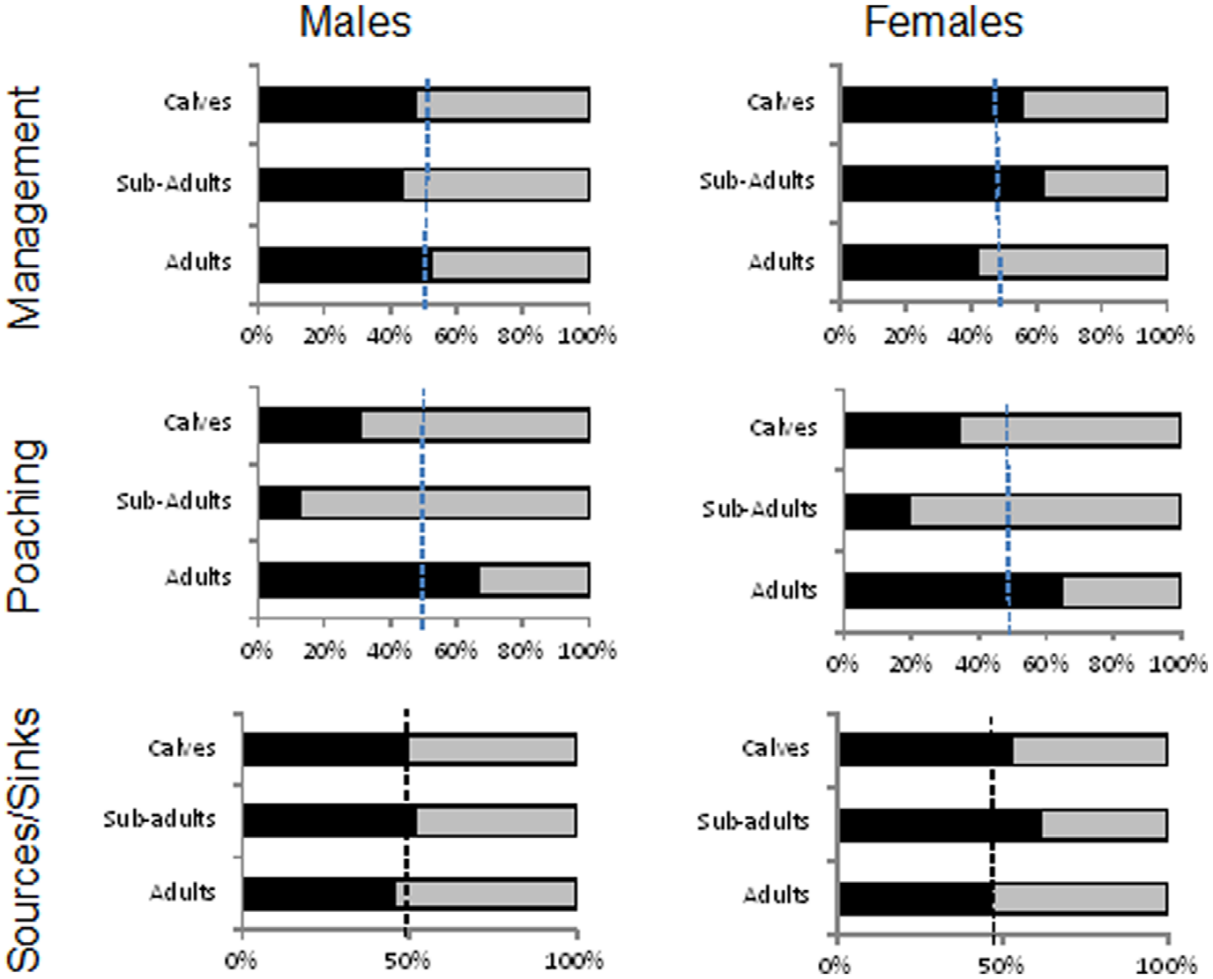
Age and sex proportions of white rhinos removed (solid bars) through management (top two graphs) during 2009 (*n_s_* = 215), 2010 (*n_s_* = 144) and 2011 (*n_s_* = 107) respectively, and poaching (bottom two graphs) during 2011 (*n_s_* = 133) in relation to age and sex proportions recorded for the population (grey bars) during 2009 (*n_s_* = 1477), 2010 (*n_s_* = 1110) and 2011 (*n_s_* = 1236) respectively. We also present age and sex proportions of white rhinos in source areas (solid bars, *n* = 2315) and sink areas (grey bars, *n* = 2725) noted during 2009–2011. If the bars separate at the vertical broken line then the proportion removed equals the proportion available.

Managers and poachers targeted different ages of rhinos – managers tend to remove a higher proportion of sub-adult females (♂: z = 1.01, p = 0.84; ♀: z = −6.42, p<0.01) than what is available, but fewer adults (♂: z = −3.65, p<0.01; ♀: z = −2.90, p<0.01) (Fig. 5). A differential effect on calves is not statistically detectable (♂: z = 0.48, p = 0.68; ♀: z = 1.06, p = 0.86). Poachers in turn targeted proportionally more adults of both sexes (calves ♂: z = −2.87, p<0.01; ♀: z = −2.87, p<0.01; sub-adults ♂: z = −4.34, p<0.01; ♀: z = −3.70, p<0.01; adults ♂: z = −2.13, p = 0.02; ♀: z = 2.54, p = 0.01). The dominant influences of management removals, however, were reflected in the comparative age and sex structure of source and sink areas. Sub-adult females made up a smaller proportion of the population in sink areas compared to elsewhere (Fig. 5).

### Landscape-specific Trends

Trends in white rhino dynamics varied substantially within landscapes (Table 2). Eight of the 35 landscapes did not have sufficient data to fit population models. In most of these cases, the landscapes have not been colonized by white rhinos since their introduction into the Park in the 1960 s. Trends in population estimates in twelve of the remaining landscapes were best associated with equilibrium models, while trends in 15 landscapes were best described by exponential models.

**Table 2:** Landscape specific model fits.

Landscape	Null Model				Exponential					Equilibrium						2010	r
	Model_i_	RSS_i_	AICc*i*	Δ_i_	Model_i_	RSS_i_	R^2^_i_	AICc*_i_*	Δ_i_	Model_i_		RSS_i_	R^2^_i_	AICc*_i_*	Δ_i_		
1	–	371108	–	–	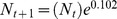	198947	0.46	143.43	13.63	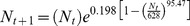		34160	0.91	122.34	0	254(179–329)	−0.127(−0.269−0.014)
2	–	352716	–	–	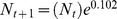	169862	0.52	141.53	0	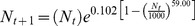		169862	0.52	141.59	0.06	673(470–876)	0.111*(0.019–0.204)
3	–	1903318	–	–	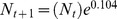	547477	0.71	155.57	0.82	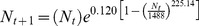		508733	0.73	154.75	0	1383(1050–1717)	0.036(−0.012–0.083)
4	–	2462753	–	–	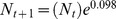	562293	0.77	155.89	10.15	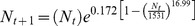		239986	0.90	145.74	0	1533(1095–1972)	−0.002(−0.020–0.024)
5	–	1658679	–	–	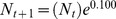	402922	0.76	151.89	2.53	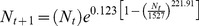		324389	0.80	149.36	0	1307(1016–1597)	0.063*(0.025–0.100)
6	–	171500	–	–	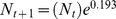	106552	0.38	135.93	0	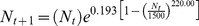		106552	0.38	136.00	0.06	335(212–459)	0.397*(0.170–0.624)
7	–	19883	–	–	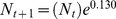	10583	0.47	108.22	11.81	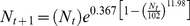		3934	0.80	96.41	0	59(22–95)	−0.095(−0.229–0.037)
8	–	2174	–	–	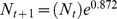	36	0.98	40.00	0	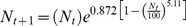		36	0.98	40.07	0.07	49(16–82)	0.677*(0.215–1.139)
9	–	31836	–	–	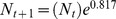	1436	0.95	84.25	0	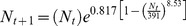		1436	0.95	84.32	0.07	192(91–293)	0.517*(0.273–0.760)
10	–	3788	–	–	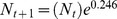	1654	0.56	85.95	0.45	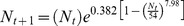		1584	0.58	85.50	0	50(0–100)	0.330*(0.148–0.511)
11	–	14017	–	–	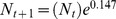	8670	0.38	105.83	20.15	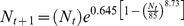		1609	0.89	85.68	0	35(17–53)	−0.178(−0.360–0.004)
12	–	61798	–	–	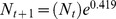	11372	0.82	109.08	0	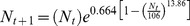		21544	0.65	116.81	7.73	267(75–459)	0.335*(0.172–0.497)
13	–	471778	–	–	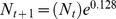	175088	0.56	141.89	1.96	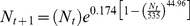		147909	0.63	139.93	0	471(342–600)	0.081(−0.124–0.287)
14	–	30982	–	–	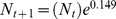	20337	0.34	116.06	0	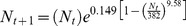	20337		0.34	116.12	0.06	126(49–203)	0.325*(0.156–0.494)
15	–	–	–	–	–	–	–	–	–	–	–		–	–	–	12(0–24)	–
16	–	50962	–	–	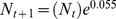	49663	0.03	126.77	5.20	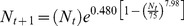	32020		0.37	121.57	0	0	−0.953*(−1.511– −0.395)
17	–	1767507	–	–	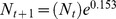	241856	0.86	145.77	6.83	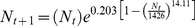	136141		0.92	138.94	0	1249(958–1540)	0.102*(0.046–0.159)
18	–	251336	–	–	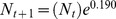	76340	0.70	131.93	19.78	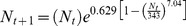	14614		0.94	112.16	0	317(177–456)	−0.029*(−0.050– −0.008)
19	–	718275	–	–	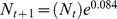	424613	0.41	152.52	0	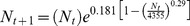	425523		0.41	152.61	0.09	909(646–1171)	0.038(−0.096–0.172)
20	–	307840	–	–	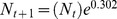	88028	0.71	133.64	0.12	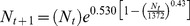	86681		0.72	133.52	0	486(267–705)	0.389*(0.094–0.683)
21	–	8263	–	–	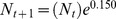	5627	0.32	100.64	0.89	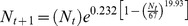	5196		0.37	99.75	0	47(22–72)	0.048(−0.029–0.124)
22	–	12447	–	–	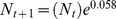	11461	0.08	109.18	0	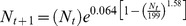	11461		0.08	109.24	0.06	54(19–89)	0.311(−0.550–1.172)
23	–	222504	–	–	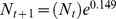	133189	0.40	138.61	27.44	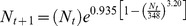	13462		0.94	111.17	0	211(110–313)	−0.113(−0.287–0.060)
24	–	6763	–	–	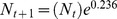	3954	0.42	96.41	2.95	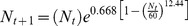	3075		0.55	93.45	0	45(10–80)	0.860*(0.351–1.368)
25	–	–	–	–	–	–	–	–	–	–	–		–	–	–	0	–
26	–	–	–	–	–	–	–	–	–	–	–		–	–	–	0	–
27	–	727	–	–	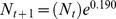	497	0.32	71.51	13.37	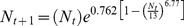	162		0.78	58.13	0	16(0–32)	−0.031(−0.588–0.525)
28	–	–	–	–	–	–	–	–	–	–	–		–	–	–	0	–
29	–	672266	–	–	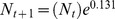	332848	0.50	149.60	10.67	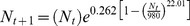	135871		0.80	138.91	0	424(293–556)	−0.009(−0.171–0.153)
30	–	–	–	–	–	–	–	–	–	–	–		–	–	–	0	–
31	–	8725	–	–	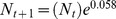	8303	0.05	105.31	0	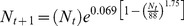	8303		0.05	105.37	0.06	27(0–54)	0.211(−0.126–0.549)
32	–	–	–	–	–	–	–	–	–	–	–		–	–	–	0	–
33	–	–	–	–	–	–	–	–	–	–	–		–	–	–	0	–
34	–	–	–	–	–	–	–	–	–	–	–		–	–	–	0	–
35	–	1890	–	–	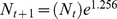	114	0.94	53.89	0.24	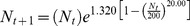	112		0.94	53.65	0	46(0–91)	0.561(−0.100–1.222)

Differences in population growth within landscapes during 2006 to 2010 were primarily associated with abundance and the number of rhinos removed by managers (Table 3). The lack of suitable landscape-specific poaching data constrained our analysis, but the relative little influence of detectable poaching effects on population scales negates this shortcoming. Density-dependence made very little contribution to explain variance in landscape-specific white rhino population growth rates.

**Table 3:** Associations of deviances in population growth in landscapes.

Model variables	*R^2^_i_*	*AICc_i_*	*Δ_i_*	*w_i_*
Abundance	0.22	−98.66	0.14	0.31
Removals	0.23	−98.81	–	0.33
Density-dependence	0.02	−92.48	6.32	0.01
Abundance + Removals	0.24	−96.83	1.97	0.12
Abundance + Density-dependence	0.24	−96.12	2.69	0.09
Removals + Density-dependence	0.23	−96.29	2.51	0.09
Abundance + Removals +Density-dependence	0.24	−94.04	4.76	0.03

### Predicted Anthropogenic Effects

**Figure 6:**
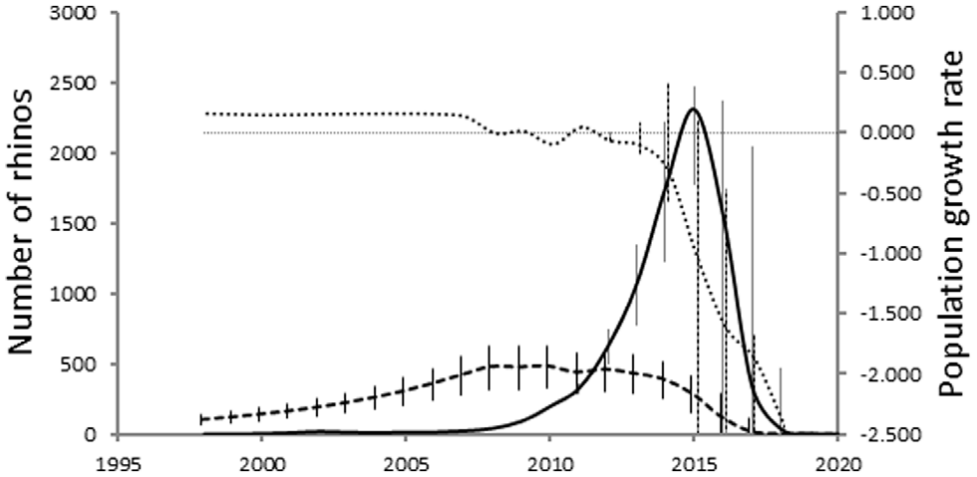
Number of rhinos predicted from the harvesting model for removal (broken line) and predictions for the likely number of rhinos poached (solid line), as well as the resultant instantaneous exponential population growth (stippled line). The error bars represent 95% confidence intervals. The horizontal fine line is population growth of zero.

SANParks’ rhino management model predicts that rhinos should be removed at a rate of 4.4% (95% CI: 0.9–7.8%) of the standing population size at any time. The present trend in the proportion of rhinos poached predicts an annual exponential rate of increase of 0.60 (95% CI: 0.55–0.66). Simulation results suggest that between 2011 and 2012, the number of rhinos poached will equal the number required to be removed for management purposes. The number of rhinos poached will exceed management requirements by 2013. If poaching continues then the population will decline significantly by 2016 (confidence intervals exclude zero) although point estimates of population growth are already consistently below zero by 2013. At that time between 505 and 735 rhinos may be poached annually (Fig. 6).

## Discussion

Large scale exploitation of wildlife resources threatens several species’ populations globally [6]. Our analyses of population dynamics and influences of anthropogenic removals of white rhinos in Kruger National Park, the largest population in the world, suggest that poaching has already compromised some conservation values and may soon compromise the persistence of the population itself. The population may be fluctuating non-directionally as a result of white rhino removals and not density-dependent processes; poachers now remove as many rhinos as what management models seek to remove, but they target adult rhinos; and continued trends in poaching may lead to detectable population declines as soon as 2016. These findings about population trends rely heavily on the precision of rhino estimates. In our case, three different estimators vary and are of some concern.

Estimating abundances of species in a particular area of interest carries large challenges. This is because several sources of error prevent conservationists from obtaining exact counts [40], [41]. For this reason, conservationists make use of numerous techniques including strip-transects [36], block counts [33], distance sampling [32], dung counts [42], mark-recapture techniques [41], call-up surveys [43], registration studies [44] and total counts [25].

Using aerial observation platforms is a common approach for estimating population sizes of large mammals [40], [45]. Hundred percent coverage of an area is usually referred to as a total count. This inherently assumes that it is a near exact estimate of the number of individuals of a specific species. The measure of how close an estimate is to the real number of individuals in a population is referred to as accuracy. Accuracy, however, has two components – bias and precision [46]. Bias originates from several sources, but is captured in three broad types.

Availability or concealment bias results when animals are present in the landscape, but not available to be sampled [40], [45]. Detection bias results when animals are present and available, but there is considerable variation in detecting those [32]. Even though availability bias and detectability bias may be accounted for, observers have different capabilities introducing observer bias [40], [41], [47]. These three biases accumulate uncertainty and influence the second component of accuracy – precision which is the likely spread of estimates [48] given the uncertainties introduced by biases. An additional source of error comes from sampling [49] which all sample-based survey approaches are exposed to. In such instances surveyors are not covering a hundred percent of an area of interest. This is captured in standard errors of an estimate, the normal statistical description of a mean and the distribution of data that supports that mean [48].

White rhino population estimates that we collated suffer at least from detectability bias – the lower estimates of 400 m strip transects result from detectability decreasing with distance from flight paths [32]. Strip-transects 200 m wide do not suffer from a similar bias and resulted in estimates similar to distance sampling estimates which corrects for detectability bias [32]. Given the values in the time series of estimates and the overlapping estimates of 200 m strip-transects, distance sampling and the one-off block count in 2009, we are confident that the trend defined by averaging these estimators best present the trends in white rhino population size within Kruger. The influence of observer bias and availability bias is unknown. However, for black rhinos these were estimated in 2009 and resulted in black rhinos being available for 90.3% of the time to be observed, but that observers will miss 3.8% of those [33]. Observer bias is likely to be the same for white rhinos, but availability bias may be different – a higher proportion of white rhinos will be available simply because of their preferences for habitats with less woody cover [50] compared to those which black rhinos prefer [51].

The discrepancies between the fixed-wing 400 m wide strip transects and other estimators may also originate from relatively low survey intensities that ranged from 14.0% to 23.9% coverage of Kruger National Park. For elephants, survey intensities that define accurate estimates require at least 5–20% coverage, but 50% coverage for precise estimates [52]. Kruger conservation managers may benefit from definitions of optimal survey requirements for white rhinos annually given the threats posed to white rhinos at present and conduct annual estimates in that way.

Making use of robust surveys is of key importance because the challenges highlighted by the difference in estimates using different techniques dampens our estimation that 8767 to 12682 white rhinos lived in Kruger during 2010, but that these were unlikely to increase. This conclusion is vulnerable to the estimates for 2010, particularly given that the same methods disregarding the highlighted shortcomings noted declines from 2008 to 2010. The 2010 estimate may result from an anomalous count and, hence, may need to be excluded. Removing the 2010 estimate will then result in the data best explained by an exponential population model. More concerning would be if the comparable methods does not reflect an anomalous count or are immune to the biases highlighted earlier. That would suggest a dramatic decline of more than half of the population in two years. That magnitude of decline is unlikely given that the presence of a large number of conservation rangers in Kruger National Park would have detected large scale mortalities. For these reasons our model averaging may accommodate these uncertainties allowing us to conclude the most likely outcome of non-directional change in rhino abundances during recent years.

Within the above context we illustrated that rhino removal for management reasons left detectable population size effects given the relative imprecision of population estimates. During 2009 and 2010, detectably more white rhinos would have lived in Kruger if no management removals took place since 1998. We could not find a similar detectable effect for poaching primarily because the confidence intervals for white rhino population estimates are too wide.

In addition, we could find detectable population structure effects associated with white rhino management removals as areas of regular removal had fewer sub-adult cows compared to elsewhere in Kruger National Park. Indeed, managers typically removed relatively more sub-adult females in a particular year compared to what was available in the population at the time. Such selective removal may result in demographic cascades [30] and contribute to the large population size effect associated with management related removals. Selective removal may also impose evolutionary constraints [53] and have indirect long-term effects on genetic integrity of the population. Management removals thus need to reflect the standing age distribution to minimize inadvertent selective pressures.

Although we could not find detectable poaching effects on population size, we would find detectable potential poaching effects on population structure – poachers killed relatively more adults than were available in the population. Such size selection by poachers is well known for elephants [54] and ultimately induces structural as well as demographic changes that lead to critical thresholds when populations collapse rapidly [30]. Poaching may thus pose a significant threat to population persistence when populations decline to threshold levels.

What, however, is the difference between management removals and rhinos removed by poachers given that management removals influenced white rhino population dynamics substantially more than poaching did since 1998? Between 2011 and 2012, we predicted that the number of rhinos poachers will be killing equals the number of rhinos defined for removal by management wishing to mimic the outcomes of impeded ecological processes. One can argue that poachers are essentially doing management! Managers, however, remove those rhinos and use them as propagules for establishment of other rhino populations within their historical distribution range where they have been extinct for some time [55], [56]. Such management removals thus make significant contributions to the recovery of the species as a whole. The entire white rhino world population has grown to more than 20000 white rhinos because of such approaches since the 1960 s initiated and advocated by the then Natal Parks Board [50]. In recent times, white rhinos from Kruger National Park are the primary sources of most privately-owned rhinos within the white rhino historical distribution. Privately-owned white rhinos comprised about 24.1% of all white rhinos in South Africa during 2010 [57]. Such restoration opportunities are lost when poachers do management!

In the second instance, managers sell white rhinos as live entities and use revenue generated to enhance protected areas [15]. SANParks, managers of Kruger, make use of game sales to augment a Parks Development Fund that support conservation infrastructure, management and research (SANParks, personal communications). Revenue generating opportunities are thus also lost when poachers do management!

In addition, poaching is not toned by ecological management models such as that used by SANParks or adaptive feedback loops [58]. Our analyses suggest frightening trends of continued increases in white rhino poaching pressure, a trend noticed world-wide for just about any natural resource that has relatively high financial value [1], [59]. Poaching predicted for 2012 has already removed opportunities to contribute to range expansion and gain financially for Kruger National Park. We predicted that by 2016 the population itself will be declining if present trends in poaching continue. In 2009 and 2010, management in Kruger adapted and earmarked rhinos for removal after poaching effects have been accounted for as it is relatively easy to apply adaptive management to local aspects under the control of managers.

**Figure 7:**
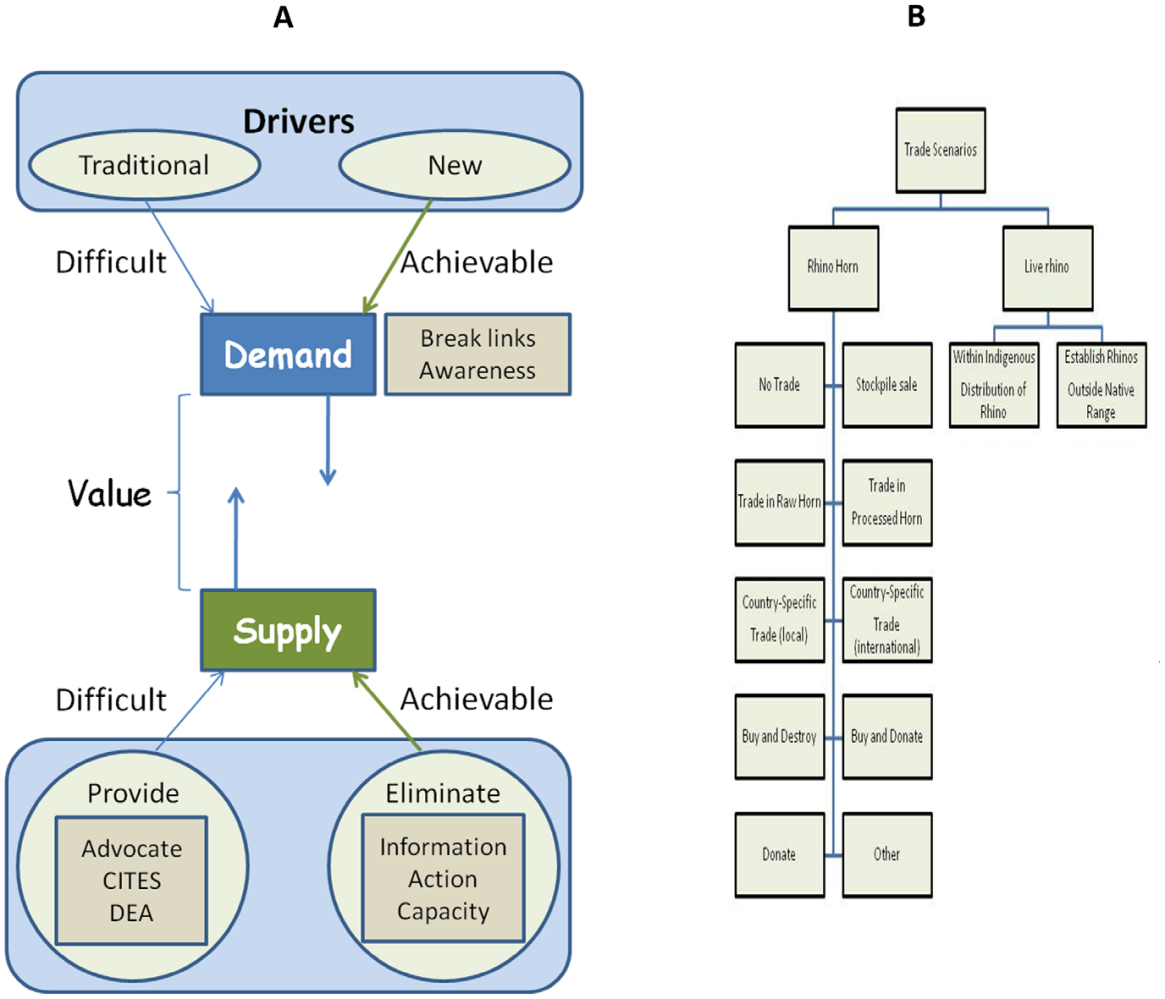
A conceptual model that defines rhino horn value and hence poaching incentives (A). We also provide examples of trade scenarios and financial models (B) that make different predictions about influences on the demand and supply ratio and ultimately white rhino population persistence.

Dealing with poaching effects is significantly more challenging because the drivers are associated with factors determining the financial value of a natural resource commodity. Demand and supply are the key underlying determinants of financial value of commodities [60]. The link between the high ratio of rhino horn demand over supply resulting in a high commodity value [9], exposes rhinos to criminal exploitation (Fig. 7A). Our results and predictions suggest that financially driven poaching incentives threatens the persistence of white rhinos as a species. Conservationists thus need to reduce the ratio of demand over rhino horn supply.

Reducing demand carries significant challenges as it faces age-old traditional inertia [3]. Although approaches are limited, strong awareness, advocacy and education campaigns may greatly contribute to reducing demand particularly if these are not associated with strong traditions such as recently claimed medicinal cures for cancer [61].

The management of supply through legalizing rhino sales carries enormous challenges (*e.g.* philosophical constraints, existing CITES international agreements and national legislation, risks to other rhino species, lack of logistical and management systems, and a need for high level political intervention). Parts of these associate with relatively limited exploration of several forms of financial models and approaches (but see [62]). By evaluating consequences of different scenarios for rhino populations as well as human livelihoods alike (Fig. 7B), international agreements such as CITES as well as national policy makers may be better informed to make decisions that curb the threats that poaching has to various conservation and societal values associated with white rhinos.

In the short to medium term, however, conservation authorities are left with eliminating supply, which is the key focus of present anti-poaching activities [57]. Our conceptual model predicts that poaching incentives should increase unless anti-poaching units can develop tactical responses that provide non-financial disincentives. For instance, low minimum wages result in little deterrent effects of fines or jail sentences [63]. In such instances, anti-poaching, often tactically re-active, carries no disincentive for a poacher to continue poaching [5]. Humanely challenging approaches, such as shoot-to-kill policies, often result [63], the effectiveness of which is uncertain. The trends that we have noted in poaching of white rhinos suggest that eliminating or reducing supply through the present anti-poaching tactics may have limited influence on poaching incentives, whether financial or non-financial.

Our results flag potential declines of the white rhino population in Kruger National Park that may be a result of poaching. We have also illustrated that at least two conservation values – sources for establishment of other populations and revenue generation – have already been compromised. We propose better surveys to define population level effects more precisely. But more importantly we advocate more pro-active tactical anti-poaching approaches already in development [57] directed at curbing poaching incursions into protected areas. Ultimately though, the international conservation community will need to find innovative ways to reduce the ratio between demand and supply that defines the financial incentives for white rhino poaching. We advocate that these challenges are shared by all exploited natural resources globally.
